# Predictability of breathing parameters during cardiopulmonary exercise testing using entropy measurements

**DOI:** 10.14814/phy2.70556

**Published:** 2025-09-29

**Authors:** Léon Genecand, Cyril Jaksic, Gaëtan Simian, Roberto Desponds, Ivan Guerreiro, Chloé Cantero, Marco Altarelli, Isabelle Frésard, Frédéric Lador, Antoine Beurnier, Pierantonio Laveneziana, David Montani, Anne Bergeron, Pierre‐Olivier Bridevaux

**Affiliations:** ^1^ Service de Pneumologie, Département de Médecine Hôpitaux Universitaires de Genève Genève Switzerland; ^2^ Faculté de Médecine Université de Genève Genève Switzerland; ^3^ Centre de Recherche Clinique Hôpitaux Universitaires de Genève Genève Switzerland; ^4^ Faculté de Mathématique Université de Genève Genève Switzerland; ^5^ Service de Pneumologie, Centre Hospitalier du Valais Romand Hôpital du Valais Sion Switzerland; ^6^ Université Paris‐Saclay, School of Medicine Le Kremlin‐Bicêtre France; ^7^ Institut National de la Santé et de la Recherche Scientifique, Unité Mixte de Recherche S_999 “Pulmonary Hypertension: Pathophysiology and Novel Therapies”, HPPIT, Marie Lannelongue Hospital Le Plessis Robinson France; ^8^ Assistance Publique‐Hôpitaux de Paris (AP‐HP), Department of Respiratory and Intensive Care Medicine, Pulmonary Hypertension National Referral Center FHU André Cournand, ERN‐LUNG, Bicêtre Hospital Le Kremlin‐Bicêtre France; ^9^ AP‐HP, Groupe Hospitalier Universitaire APHP‐Sorbonne Université, Hôpitaux Pitié‐Salpêtrière, Saint‐Antoine et Tenon Service Des Explorations fonctionnelles de la Respiration, de l'Exercice et de la Dyspnée (Département R3S) Paris France; ^10^ Sorbonne Université, INSERM, UMRS1158 Neurophysiologie Respiratoire Expérimentale et Clinique Paris France; ^11^ AP‐HP, Groupe Hospitalier Universitaire APHP‐Sorbonne Université, Hôpital Pitié‐Salpêtrière Paris France

**Keywords:** abnormal breathing pattern, approximate entropy, cardiopulmonary exercise testing, dispersion, dysfunctional breathing, sample entropy

## Abstract

Erratic breathing at rest and during exercise has been described in patients with dysfunctional breathing (DB). Sample entropy (SampEn) and approximate entropy (ApEn) were developed to assess the predictability of parameters in time series data. However, SampEn and ApEn were developed for data without trends. Their potential role in interpreting datasets during cardiopulmonary exercise testing (CPET) remains uncertain. We used simulations based on real‐life exercise data to evaluate the effects of trends, varying numbers of analyzed respiratory cycles, and different coefficients used to calculate the tolerance interval on ApEn and SampEn measurements. We tested the LOESS_0.75_ method to correct the trend in the calculation of SampEn and ApEn. Trends led to a significant underestimation of ApEn and SampEn values. The LOESS_0.75_ method yielded values very close to the predicted ApEn and SampEn. Modifying the number of respiratory cycles and the coefficient used to calculate the tolerance interval had major implications on ApEn and SampEn. In conclusion, the residuals from the LOESS_0.75_ method can provide a corrected estimate of ApEn and SampEn in exercise data. The number of respiratory cycles analyzed and the coefficient used to calculate SampEn or ApEn should always be reported.

## 1 INTRODUCTION

Dysfunctional breathing (DB) is characterized by an abnormal breathing pattern associated with compatible symptoms after ruling out an organic cause explaining these symptoms (Boulding et al., 2016). DB has been a center of interest during the SARS‐CoV‐2 pandemic, where it was thought to play an important role in symptom generation, including dyspnea (Gaffney, 2024). Different types of abnormal breathing patterns were proposed, including hyperventilation and erratic breathing, associated or not with sighs (Boulding et al., 2016). Erratic breathing is characterized by irregular breathing and large variations of tidal volume (VT). Numerous case series of patients with DB have been published after COVID infection (Beurnier et al., 2023; Fresard et al., 2022) and similar cases were already described long before the COVID‐19 pandemic (Prys‐Picard et al., 2006). Cardiopulmonary exercise testing (CPET) allows the evaluation of the main cause limiting the exercise (Laveneziana et al., 2021) and has been largely used during the COVID‐19 pandemic to investigate unexplained dyspnea after SARS‐CoV‐2 infection (Mancini et al., 2021). CPET has also been used to describe different types of DB patterns such as hyperventilation and erratic breathing (Beurnier et al., 2023; Fresard et al., 2022; Ionescu et al., 2020; Watson et al., 2021).

Although identifying erratic breathing patterns is of clinical interest, methods used so far have heavily relied on subjective assessments using visually interpreted unfiltered graphs (Beurnier et al., 2023; Fresard et al., 2022; Ionescu et al., 2020). Because of their subjectivity, these observations were criticized. The development of objective and reproducible markers of DB patterns could allow researchers to strengthen their confidence in the diagnosis of DB by establishing normative values, determining diagnostic cutoffs, and evaluating associations between DB patterns and symptoms.

**FIGURE 1:**
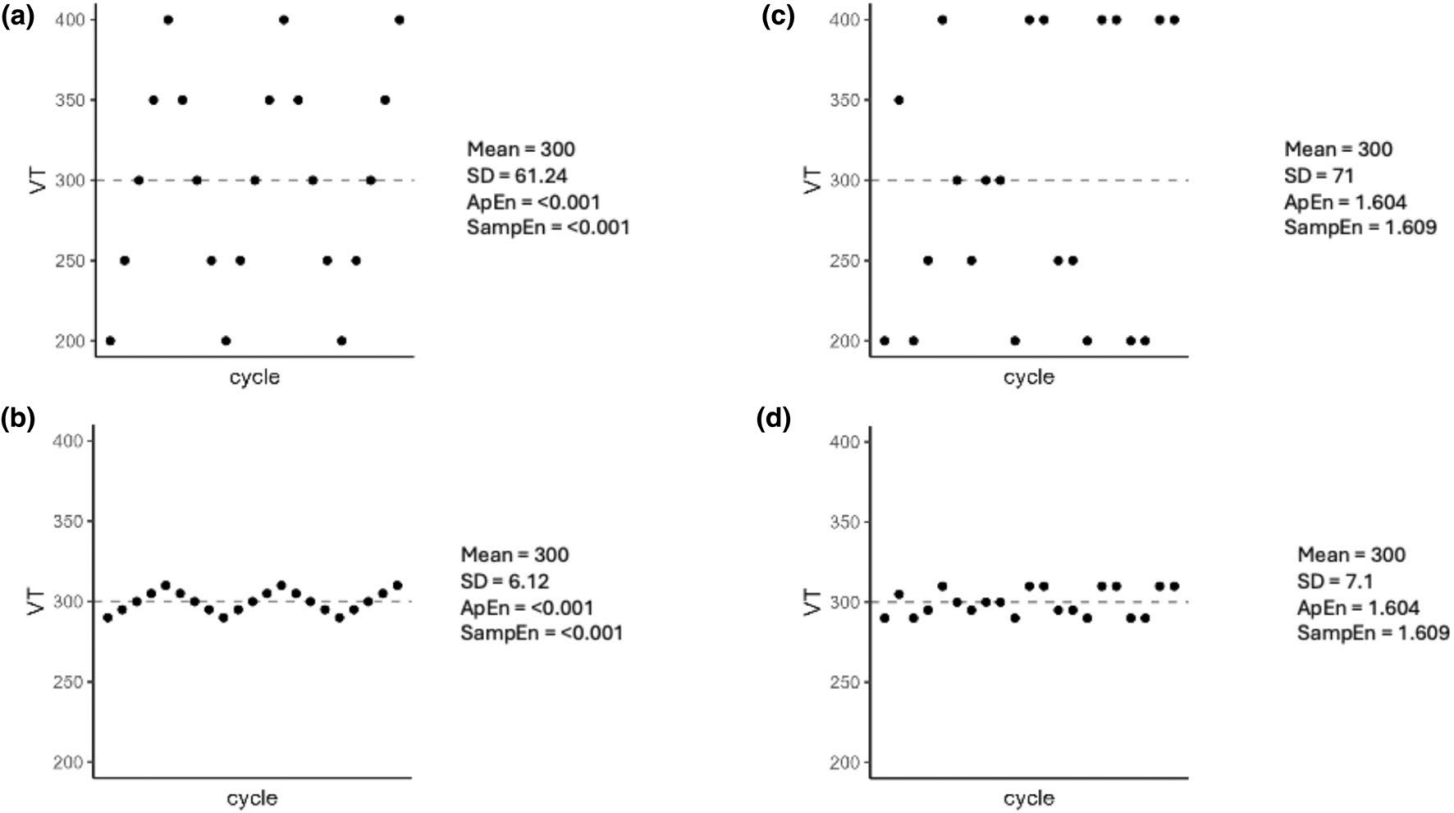
Concept of predictability and dispersion of breathing parameters. This figure illustrates the two complementary concepts of predictability and dispersion using fictive examples, showing that, in theory, all combinations of dispersion and predictability are possible: (a) high dispersion with high predictability, (b) low dispersion with high predictability, (c) high dispersion with low predictability, and (d) low dispersion with low predictability. To generate this graph, we simulated the following scenarios: a, volumes of 200, 250, 300, 350, 400, 350, 300, 250, and 200 mL were repeated following this exact order for 10,000 points; b, volumes of 290, 295, 300, 305, 310, 305, 300, 295, and 290 mL were repeated following this exact order for 10,000 points; c, volumes of 200, 250, 300, 350, and 400 mL were randomly selected for 10,000 points; d, volumes of 290, 295, 300, 305, and 310 mL were randomly selected for 10,000 points. In all scenarios, a coefficient of 0.35 was used to calculate the tolerance interval “*r*” For each scenario, we then computed the mean, SD, and entropies measurements.

We recently evaluated a method using locally estimated scatterplot smoothing (LOESS_0.75_) and showed that it could measure the dispersion around the trended value of breathing parameters during exercise with high precision and low bias in most simulated scenarios (Genecand et al., 2025). Measures of location (e.g., the mean) and of spread (e.g., standard deviation and variance) are one way to describe a dataset. Another way to describe DB could be the use of regularity statistics, based on entropy measurements, which have the ability to assess the unpredictability of tidal volumes and breathing frequencies in a time series (Delgado‐Bonal & Marshak, 2019). Both regularity statistics and measures of location and of spread can be obtained from unfiltered cycle‐by‐cycle data, where one respiratory cycle represents one data point. Figure 1 illustrates the concepts of predictability and dispersion of breathing parameters using fictive examples, showing that, in theory, all combinations of dispersion and predictability are possible: (A) high dispersion with high predictability, (B) low dispersion with high predictability, (C) high dispersion with low predictability, and (D) low dispersion with low predictability.

The concept of predictability of breathing parameters for the objective description of DB could be of potential interest and complementary to measures of location and of spread and subjective analyses of graphs. In this context, some researchers have recently used entropy measurements without correcting the trend of the data to describe DB during exercise showing some potential diagnostic abilities to discriminate normal from abnormal breathing patterns (Bansal et al., 2018; Samaranayake et al., 2023). However, the concept of entropy relies on complex mathematical calculations, and many different parameters might influence its correct interpretation.

In this article, we aimed to:

1. Show the importance of reporting measures of spread, more specifically the SD of the underlying dataset, when reporting regularity statistics.
2. Show the effect of trends during exercise on SampEn and ApEn values and offer a solution for the calculation of SampEn and ApEn when using data with trends.
3. Explain the effect of the number of respiratory cycles analyzed on the values of SampEn and ApEn and why it is important to report the number of respiratory cycles analyzed for each patient.
4. Explain the effect of the coefficient used to calculate the tolerance interval “*r*” on the values of SampEn and ApEn and why it is important to report this coefficient.

## 2 MATERIALS AND METHODS

### 2.1 Entropies measurements

Entropy measurement is a mathematical approach using nonlinear dynamical analysis or regularity statistics to evaluate chaos (i.e., “unpredictability”) in a time series dataset (Pincus, 1991; Richman & Moorman, 2000). Entropy measurements include, among others, SampEn and ApEn (Pincus, 1991; Richman & Moorman, 2000). We will only briefly describe the basis of both methods. Complete explanations, including development, tutorial for use, and computation formula of SampEn and ApEn are detailed elsewhere (Delgado‐Bonal & Marshak, 2019; Pincus, 1991; Richman & Moorman, 2000). From a mathematical point of view, SampEn (*m*, *r*, *N*) is the negative natural logarithm of the conditional probability that two sequences similar for “*m*” points remain similar at the next point (i.e., within the tolerance interval “*r*”) where self‐matches are not included in calculating the probability (Richman & Moorman, 2000). ApEn is based on the same mathematical principle as SampEn, but self‐matches are included in calculating the probability (Pincus, 1991). To calculate whether sequences remain close or are within the tolerance interval, the distance between the highest point and lowest point of the analyzed data sequence on the Y axis is calculated (Richman & Moorman, 2000). Since the amplitude of this difference does not count in the calculation but rather the probability that this amplitude is out of the tolerance interval or stays within, SampEn and ApEn do not give a measure of the dispersion of data (Delgado‐Bonal & Marshak, 2019). For both entropy methods, different parameters must be specified before calculation. These are “*N*”, the overall amount of data in the analyzed time series, “*m*” the length of the analyzed sequences within the time series, and the “coefficient” used to calculate “*r*”, the tolerance interval for accepted matches. The coefficient used to calculate “*r*” that maximizes the value of ApEn has been suggested to be the most suitable value for ApEn calculation (Delgado‐Bonal & Marshak, 2019). Default settings offered by R statistical software for SampEn and ApEn include m = 2 and *r* = 0.2*standard deviation (SD). “*N*” is fixed by the sequence being analyzed. During CPET, “*N*” is fixed by the number of respiratory cycles when using unfiltered (i.e., cycle‐by‐cycle) data of breathing parameters. Because SampEn and ApEn are based on the logarithm of probabilities, and probabilities take any value between 0 and 1, SampEn and ApEn can, in theory, take any value between 0 and an infinite number for infinite “*N*” and infinite possible combinations of numbers. Higher values of SampEn and ApEn represent more “unpredictable” data. For the calculation of SampEn and ApEn, we have used *m* = 2 (Pincus, 1991; Richman & Moorman, 2000). This article will not detail the effect of changing the parameter “*m*” and further explanations can be found elsewhere (Delgado‐Bonal & Marshak, 2019). Generally, using the default parameter *m* = 2 is probably reasonable for the type of data analyzed during CPET because of the relatively small amount of data (Delgado‐Bonal & Marshak, 2019). Changing “*m*” will significantly impact entropies calculations, and therefore reporting is mandatory (Delgado‐Bonal & Marshak, 2019).

### 2.2 Simulations used

In this article, we used simulations previously published that evaluated the performance of the moving standard deviation (MSD) and the LOESS to describe the dispersion of data around a trended parameter. The full method and results of the simulations have been published (Genecand et al., 2025). Briefly, the simulations were based on real‐life data from a case series of patients diagnosed with DB after SARS‐CoV‐2 infection (Genecand et al., 2023). This case series was used to set the target simulated parameters to ensure that the simulated scenarios were plausible with real‐life exercise data. Simulations were used because this process allows to know the true parameters, which is not possible with real‐life data (Morris et al., 2019). We first simulated data with no trend with a known dispersion of the data (for BF SD of 2/min, 4/min, and 6/min; for VT SD of 60 mL, 270 mL, and 500 mL). A trend was applied to the simulated data to mimic exercise. We then tested two methods (MSD and LOESS) and calculated their bias (i.e., the difference between the mean value of the dispersion of either VT or BF obtained with the tested method and the true dispersion) and their precision (i.e., SD of the dispersion of either VT or BF as measured by the tested method). These simulations showed that the LOESS was globally more precise and had lower bias than the MSD in most simulated exercise scenarios (Genecand et al., 2025). We therefore concluded that the dispersion of breathing parameters can be described using the LOESS with a span of 0.75 (LOESS_0.75_) in most scenarios with very high precision and low bias.

### 2.3 Correcting trends for entropy measurements

The LOESS method is one of the numerous statistical methods that can be used to suppress the trend of data. Once the nonparametric regression line of the LOESS has been fitted, residuals are automatically generated. The residuals represent the vertical differences between each point and the LOESS regression line. If the match of the LOESS to the dataset is good, the residuals will be centered on 0 and the SD of the residuals will closely represent the dispersion around the trended parameter. In this context, the residuals also represent the dataset after trend suppression. Residuals can also be used to calculate the entropy after suppression of the trend in the dataset. Figure 4 of the previously published article shows in more detail how the LOESS works (Genecand et al., 2025).

### 2.4 Evaluation of the effect of the number of respiratory cycles, the coefficient used to calculate *r*, and the trend on entropy measurements

In the present article, we used the simulations of BF and VT previously published, except the simulations with sighs, because assessing the effect of outliers on entropy measurements was beyond the scope of the present study. We therefore used 6 of the 9 simulation scenarios (3 simulations for VT and 3 simulations for BF) (Genecand et al., 2025). To evaluate entropy's properties, we used an “*N*” (i.e., number of respiratory cycles) fixed at 300 in most simulations because it approximates the average number of respiratory cycles from the case series used to create the simulations (Genecand et al., 2023, 2025). “*N*” only differed from 300 in simulations specifically evaluating the effect of varying “*N*”. The parameter “*m*” was fixed at 2. We then:

1. measured the coefficient of the tolerance interval “*r*” where ApEn was maximized by using the (ApEn – SampEn)/(coefficient of “*r*”) relationship in the simulated scenarios.
2. measured both SampEn and ApEn using the coefficient of the tolerance interval “*r*” that maximized the value of ApEn in simulations without trends.
3. tested the effect of the trend on SampEn and ApEn measurements using the residuals obtained with the LOESS_0.75_ method and compared the simulated values of SampEn and ApEn with their values after the trend was applied.
4. evaluated the ApEn‐SampEn over “*N*” relationship in the simulated scenarios by repeating the same simulations with various “*N*”.

### 2.5 Examples in two real‐life examples focusing on the effect of the trends

We used cycle‐by‐cycle data from two patients diagnosed with DB from our previously published study (Genecand et al., 2023) to illustrate how entropy measurements can be displayed during CPET, how the SD_trend_/SD_true_ ratio can be calculated and visualized, and to highlight its impact. For this, we used the raw cycle‐by‐cycle VT data, calculated “*N*” (the number of respiratory cycles included in the entropy computation), and determined the mean and standard deviation (SD) of the raw cycle‐by‐cycle dataset, which reflects the SD_trend_. We then applied a LOESS regression to the data to obtain residuals, allowing us to compute the dispersion around the trend (i.e., SD_true_). The SD_trend_/SD_true_ ratio was subsequently calculated. ApEn_0.2_ and SampEn_0.2_ were computed on both the raw and detrended cycle‐by‐cycle data. The coefficient used to calculate the tolerance interval was fixed at 0.2 as suggested to be an acceptable parameter for the analysis of most real‐life data (Delgado‐Bonal & Marshak, 2019).

## 3 RESULTS

### 3.1 General explanations of the dispersion measurement using the LOESS_0.75_

Table 1 presents the simulations for BF with simulated dispersions (i.e., SD Flat) of 2/min, 4/min, and 6/min. Table 2 presents the simulations for VT with simulated dispersions (i.e., SD Flat) of 60 mL, 270 mL, and 500 mL. Trends are then applied to the simulations, using linear and exponential (e40) trends for BF and linear and logarithmic (ln) trends for VT. Calculating the dispersion using the LOESS_0.75_ is expected to yield exactly the simulated dispersion for all trends.

**TABLE 1:** Simulations of BF using a SD of 2/min, 4/min, and 6/min.

Methods	Flat (no trend)	Linear trend	Exponential trend (e40)
Simulated SD of 2			
SD	2 (±0)	6.39 (±0.11)	5.12 (±0.11)
LOESS_0.75_	1.99 (±0.01)	1.99 (±0.01)	2.05 (±0.03)
ApEn	1.33 (±0.03)	0.77 (±0.03)	0.85 (±0.03)
SampEn	1.64 (±0.06)	0.75 (±0.04)	0.82 (±0.04)
ApEn measured on the LOESS_0.75_ residuals	1.33 (±0.03)	1.33 (±0.03)	1.32 (±0.03)
SampEn measured on the LOESS_0.75_ residuals	1.64 (±0.06)	1.65 (±0.06)	1.64 (±0.06)
Simulated SD of 4			
SD	4 (±0)	7.27 (±0.19)	6.18 (±0.18)
LOESS_0.75_	3.97 (±0.02)	3.97 (±0.02)	4.01 (±0.03)
ApEn	1.33 (±0.03)	1.13 (±0.03)	1.16 (±0.03)
SampEn	1.64 (±0.06)	1.23 (±0.05)	1.28 (±0.06)
ApEn measured on the LOESS_0.75_ residuals	1.33 (±0.03)	1.33 (±0.03)	1.33 (±0.03)
SampEn measured on the LOESS_0.75_ residuals	1.64 (±0.06)	1.65 (±0.06)	1.64 (±0.06)
Simulated SD of 6			
SD	6 (±0)	8.54 (±0.25)	7.63 (±0.21)
LOESS_0.75_	5.96 (±0.03)	5.96 (±0.03)	5.98 (±0.04)
ApEn	1.33 (±0.03)	1.24 (±0.04)	1.25 (±0.04)
SampEn	1.64 (±0.06)	1.45 (±0.06)	1.46 (±0.06)
ApEn measured on the LOESS_0.75_ residuals	1.33 (±0.03)	1.33 (±0.03)	1.33 (±0.03)
SampEn measured on the LOESS_0.75_ residuals	1.64 (±0.06)	1.65 (±0.06)	1.65 (±0.06)

*Note*: The data were simulated randomly with a normal distribution. Data are calculated using mean (±SD). ApEn measurement on the LOESS_0.75_ residuals corresponds to ApEn measurement after the trend has been corrected; SampEn measurement on the LOESS_0.75_ residuals corresponds to SampEn measurement after the trend has been corrected. ApEn and SampEn values were calculated using a *N* of 300, *m* of 2, and a coefficient used to calculate “*r*” of 0.35 (i.e., the coefficient that maximized the value of ApEn) in the simulations.

Abbreviations: ApEn, approximate entropy; LOESS_0.75_, locally estimated scatterplot smoothing with a span of 0.75; SampEn, sample entropy.

**TABLE 2:** Simulations of tidal volume without sighs.

Methods	Flat (no trend)	Linear trend	Logarithmic trend (ln)
SD of 60 (no sighs)			
SD	60 (−)	392.1 (±3.4)	234.65 (±3.35)
LOESS_0.75_	59.6 (±0.3)	59.6 (±0.3)	73.6 (±1.9)
ApEn	1.33 (±0.03)	0.3 (±0.01)	0.6 (±0.02)
SampEn	1.64 (±0.06)	0.3 (±0.01)	0.57 (±0.03)
ApEn measured on the LOESS_0.75_ residuals	1.33 (±0.03)	1.33 (±0.03)	1.28 (±0.03)
SampEn measured on the LOESS_0.75_	1.64 (±0.06)	1.65 (±0.06)	1.48 (±0.06)
SD of 270 (no sighs)			
SD	270 (−)	472.2 (12.8)	352.6 (±10.04)
LOESS_0.75_	268.1 (±1.2)	268.1 (±1.2)	271.6 (±2.6)
ApEn	1.33 (±0.03)	1.15 (±0.03)	1.25 (±0.03)
SampEn	1.64 (±0.06)	1.27 (±0.05)	1.47 (±0.06)
ApEn measured on the LOESS_0.75_ residuals	1.33 (±0.03)	1.33 (±0.03)	1.32 (±0.03)
SampEn measured on the LOESS_0.75_	1.64 (±0.06)	1.65 (±0.06)	1.64 (±0.06)
SD of 500 (no sighs)			
SD	500 (−)	632.5 (±17.8)	549.1 (±11.9)
LOESS_0.75_	496.5 (±2.3)	496.5 (±2.3)	498.4 (±3.3)
ApEn	1.33 (±0.03)	1.28 (±0.04)	1.31 (±0.04)
SampEn	1.64 (±0.06)	1.54 (±0.06)	1.6 (±0.06)
ApEn measured on the LOESS_0.75_ residuals	1.33 (±0.03)	1.33 (±0.03)	1.33 (±0.03)
SampEn measured on the LOESS_0.75_	1.64 (±0.06)	1.65 (±0.06)	1.64 (±0.06)

*Note*: Simulation of VT using a SD of 60 mL, 270 mL, and 500 mL. The data were simulated randomly with a normal distribution (no sighs). Data are calculated using mean (±SD). ApEn measurement on the LOESS_0.75_ residuals corresponds to ApEn measurement after the trend has been corrected; SampEn measurement on the LOESS_0.75_ residuals corresponds to SampEn measurement after the trend has been corrected. ApEn and SampEn values were calculated using a *N* of 300, *m* of 2, and a coefficient used to calculate “*r*” of 0.35 (i.e., the coefficient that maximized the value of ApEn) in the simulations.

Abbreviations: ApEn, approximate entropy; LOESS_0.75_, locally estimated scatterplot smoothing with a span of 0.75; SampEn, sample entropy.

For example, in the simulations of BF with an SD of 2/min (Table 1), the LOESS_0.75_ measures a mean dispersion (±SD) of 1.99 (±0.01) after a linear trend has been applied and 2.05 (±0.03) after an exponential trend has been applied. The bias of the LOESS_0.75_ method can be calculated as the average dispersion minus the simulated (i.e., true) dispersion. In the case of an exponential trend, the bias is 0.05 (i.e., 2.05–2), and the precision, defined as the SD of the measurement, is (±0.03). Similar reasoning applies to all simulations.

On the other hand, simply calculating the SD of the dataset after a trend has been applied does not measure the dispersion around the trend and is significantly influenced by the trend itself. For example, calculating the SD for data where an SD of 2/min was simulated and a linear trend was applied resulted in an SD of 6.39 (±0.11).

### 3.2 Entropy measurement, effect of the trend and of the dispersion

The mean SampEn and ApEn values for data with no trend (flat) were 1.64 and 1.33, respectively, across the six simulations, as shown in Tables 1 and 2, and did not vary with the simulated dispersion. After applying a trend, the SampEn and ApEn values systematically decreased with varying amplitudes (shown in Tables 1 and 2). For example, after applying a linear trend to the simulation of BF with a dispersion of 2/min, ApEn values dropped from 1.33 (±0.03) to 0.77 (±0.03) (Table 1). For the same trend but with simulated dispersions of 4/min and 6/min, ApEn values dropped from 1.33 (±0.03) to 1.13 (±0.03) and 1.24 (±0.04), respectively. Interestingly, the decrease in ApEn is lower for a given trend when the simulated dispersion is larger. As shown in Tables 1 and 2, this observation is true for both BF and VT simulations and for all applied trends.

**FIGURE 2:**
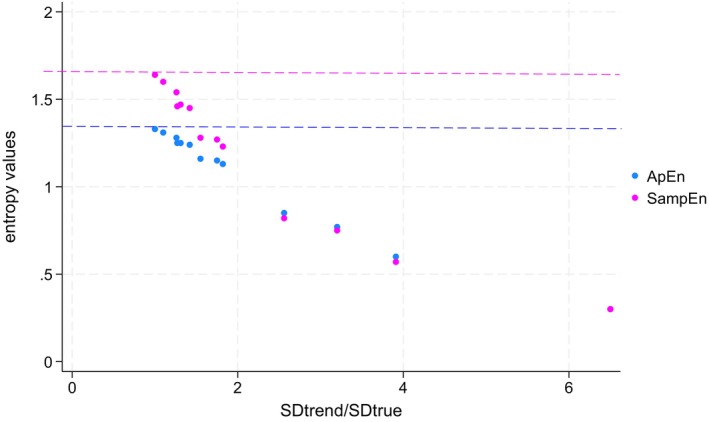
Errors in entropy estimations (ApEn and SampEn) induced by the trend. ApEn, SampEn are obtained from Tables 1 and 2. ApEn and SampEn are plotted against the SD_trend_/SD_true_ ratio. The blue line is set at 1.33, which is the predicted value of ApEn. The magenta line is set at 1.64, which is the predicted value of SampEn. As explained in the text, from a mathematical point of view, neither ApEn nor SampEn should decrease when a trend is applied. However, both ApEn and SampEn decrease when a trend is applied. The magnitude of the effect is explained by the ratio SD_true_/SD_trend_. Therefore, simply calculating ApEn and SampEn in the presence of a trend does not represent the true predictability of the data set.

Examining Table 1 in greater detail, the largest drop in entropy for BF occurs when a linear trend is applied to a simulated dispersion of 2/min, where ApEn values decline from 1.33 (±0.03) to 0.77 (±0.03). The smallest drop in entropy occurs when an exponential trend is applied to a simulated dispersion of 6/min, where ApEn values decrease from 1.33 (±0.03) to 1.25 (±0.04). Notably, the largest drop (from 1.33 ± 0.03 to 0.77 ± 0.03) corresponds to the highest ratio between the SD measured after the trend is applied (SD_trend_) and the simulated SD (SD_true_). In this example, the ratio SD_trend_/SD_true_ is 6.39/2 = 3.20. Conversely, the smallest drop in ApEn (from 1.33 ± 0.03 to 1.25 ± 0.04) is associated with the smallest ratio between SD_trend_ (7.63) and SD_true_ (6), given by 7.63/6 = 1.27. In fact, the decrease in both ApEn and SampEn can be predicted by the ratio between SD_trend_ and SD_true_ across all scenarios. This relationship is illustrated in Figure 2, which presents ApEn and SampEn values according to the SD_trend_/SD_true_ ratio.

### 3.3 Use of the LOESS_0.75_ to correct for the effect of the trend for entropy measurement

**FIGURE 3:**
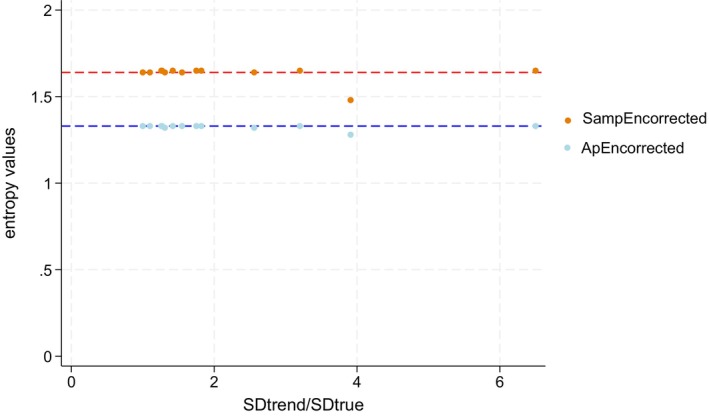
Correction in entropy estimations (ApEn and SampEn) using the residual of the LOESS_0.75_. SampEn and ApEn calculated on the LOESS_0.75_ residuals are obtained from Tables 1 and 2. SampEn and ApEn corrected using the LOESS_0.75_ method (ApEn measured on the LOESS_0.75_ residuals and SampEn measured on the LOESS_0.75_ residuals) are plotted against the SD_trend_/SD_true_. The blue line is set at 1.33, which is the predicted value of ApEn. The orange line is set at 1.64, which is the predicted value of SampEn. This graph shows that SampEn measured on the LOESS_0.75_ residuals and ApEn measured on the LOESS_0.75_ residuals are close to the predicted value of the entropy in most simulated scenarios. We observe two points, one for ApEn measured on the LOESS_0.75_ residuals and one for SampEn measured on the LOESS_0.75_ residuals, that do not align with their respective predicted values (predicted ApEn = 1.33; predicted SampEn = 1.64). These two points correspond to the simulation of VT with an SD of 60 and a logarithmic trend. ApEn_corrected_, ApEn measured on the LOESS_0.75_ residuals; SampEn_corrected_, SampEn measured on the LOESS_0.75_ residuals.

As explained before, the LOESS_0.75_ method can be used to suppress the trend by using the residuals of its measurement. As can be observed in Tables 1 and 2, when the SampEn is calculated on the residuals of LOESS_0.75_ and the ApEn on the residuals of the LOESS_0.75_, the results are very close to the measured entropy on the simulated data (i.e., the true entropy of the dataset). For example, the ApEn on the LOESS_0.75_ residuals calculated after a linear trend was applied to a simulated dispersion of 2/min is 1.33 (±0.03), identical to the ApEn calculated on the initial data before the trend was applied. Figure 3 plots the values of SampEn calculated on the LOESS_0.75_ residuals and ApEn calculated on the LOESS_0.75_ residuals over SD_trend_/SD_true_ values, demonstrating that entropy values calculated using this method closely match the predicted entropy values in most scenarios.

**FIGURE 4:**
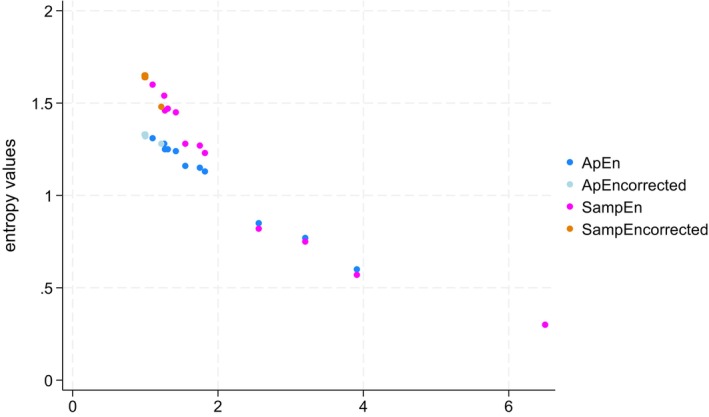
Small errors in entropy estimations using ApEn and SampEn measured on the LOESS_0.75_ residuals are caused by the inability of the LOESS_0.75_ to perfectly correct for the trend. ApEn and SampEn are plotted against the SDtrend/SDtrue ratio (x‐axis), while ApEn and SampEn measured on the LOESS_0.75_ residuals (i.e., ApEn and SampEn corrected) are plotted against the SD_loess0.75_/SDtrue ratio (x‐axis). The predicted values are 1.33 for ApEn and 1.64 for SampEn. We observe two points, one for ApEn measured on the LOESS_0.75_ residuals and one for SampEn measured on the LOESS_0.75_ residuals, that do not align with their respective predicted values (predicted ApEn = 1.33; predicted SampEn = 1.64). These two points correspond to the highest SD_loess0.75_/SDtrue ratio of 1.23 (73.6/60) in the simulation of VT with an SD of 60 and a logarithmic trend. This trend results in an estimated dispersion of 73.6 when using the LOESS_0.75_ method. The corresponding values of SampEn measured on the LOESS_0.75_ residuals and ApEn measured on the LOESS_0.75_ residuals are 1.48 (±0.06) and 1.28 (±0.03), respectively, yielding the two observed points. All other values are so close to the predicted values using LOESS_0.75_ that they are indistinguishable from their predicted values. Similar to ApEn and SampEn, the slight drop in ApEn measured on the LOESS_0.75_ residuals and SampEn measured on the LOESS_0.75_ residuals for the simulation of VT with an SD of 60 and a logarithmic trend is explained by the SD_loess0.75_/SDtrue ratio. Therefore, the error in entropy calculations using the residuals of LOESS_0.75_ arises from the LOESS_0.75_ method's own limitations in correcting for the trend. ApEn_corrected_, ApEn measured on the LOESS_0.75_ residuals; SampEn_corrected_, SampEn measured on the LOESS_0.75_ residuals.

However, in certain cases, the ApEn and SampEn values differed from the simulated values. For instance, in Table 2, for the simulations of VT with a dispersion of 60 mL after a logarithmic trend was applied, the ApEn measured on the LOESS_0.75_ residuals yielded a value of 1.28 (±0.03), while the calculated value on the simulated dataset was 1.33 (±0.03). The remaining error in entropy calculation using the corrected method with the LOESS_0.75_ residuals is attributed to the bias and imprecision of the LOESS_0.75_ method itself. This is illustrated by overlaying ApEn and SampEn values over the SD_trend_/SD_true_ ratio and ApEn measured on the LOESS_0.75_ residuals and SampEn measured on the LOESS_0.75_ residuals values over the SD_LOESS0.75_/SD_true_ ratio, where the SD_LOESS0.75_ corresponds to the SD calculated using the LOESS_0.75_ method after applying a trend to the simulated dataset. This is shown in Figure 4.

### 3.4 Effect of the coefficient of “*r*” on entropy measurement

**FIGURE 5:**
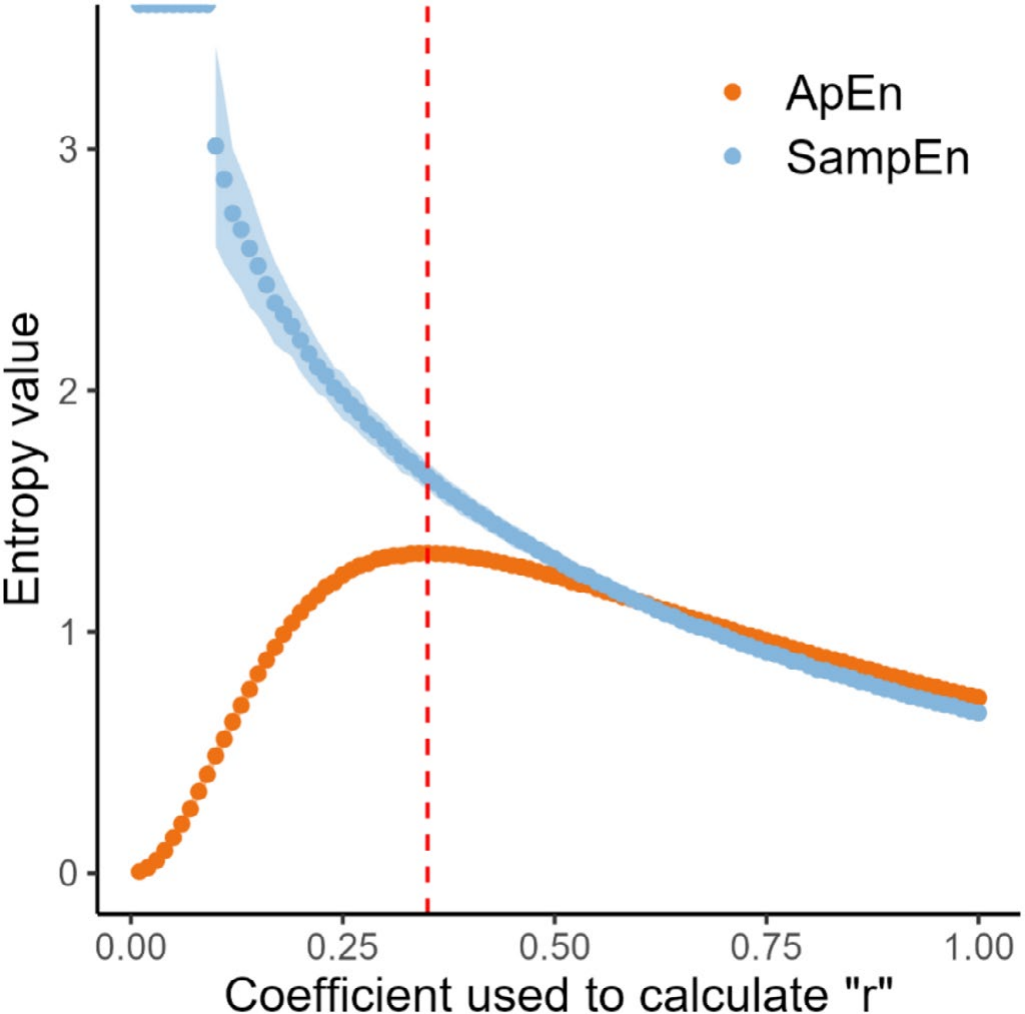
The ApEn and SampEn over the coefficient of “*r*” relationship is shown graphically for one simulation (simulations of VT with a mean of 790 and a SD of 270). To generate this graph, we used similar methodology as Richman and Moorman (2000) but applied to our simulations. The value of the coefficient that maximized the value of “*r*” was 0.35. The value of SD had no influence on the ApEn and SampEn over the coefficient used to calculate “*r*” relationships, which was similar across all simulations (not shown). Blue ribbons represent the SD of the SampEn measurement (precision of the measurement). Orange ribbons are not visible because ApEn measurement had virtually no measurable imprecision.

Figure 5 shows the evolution of SampEn and ApEn for VT when the coefficient used to calculate the tolerance interval, “*r*”, is modified. SampEn decreases as higher values of the coefficient for “*r*” are applied. ApEn, on the other hand, follows a bell‐shaped pattern, peaking at a coefficient of 0.35, which maximizes its value. Other simulations of VT, as well as simulations of BF using different dispersion values, yielded the exact same (ApEn – SampEn)/(coefficient of ‘*r*’) relationship (data not shown).

### 3.5 Effect of the number of respiratory cycles “*N*” on entropy measurement

**FIGURE 6:**
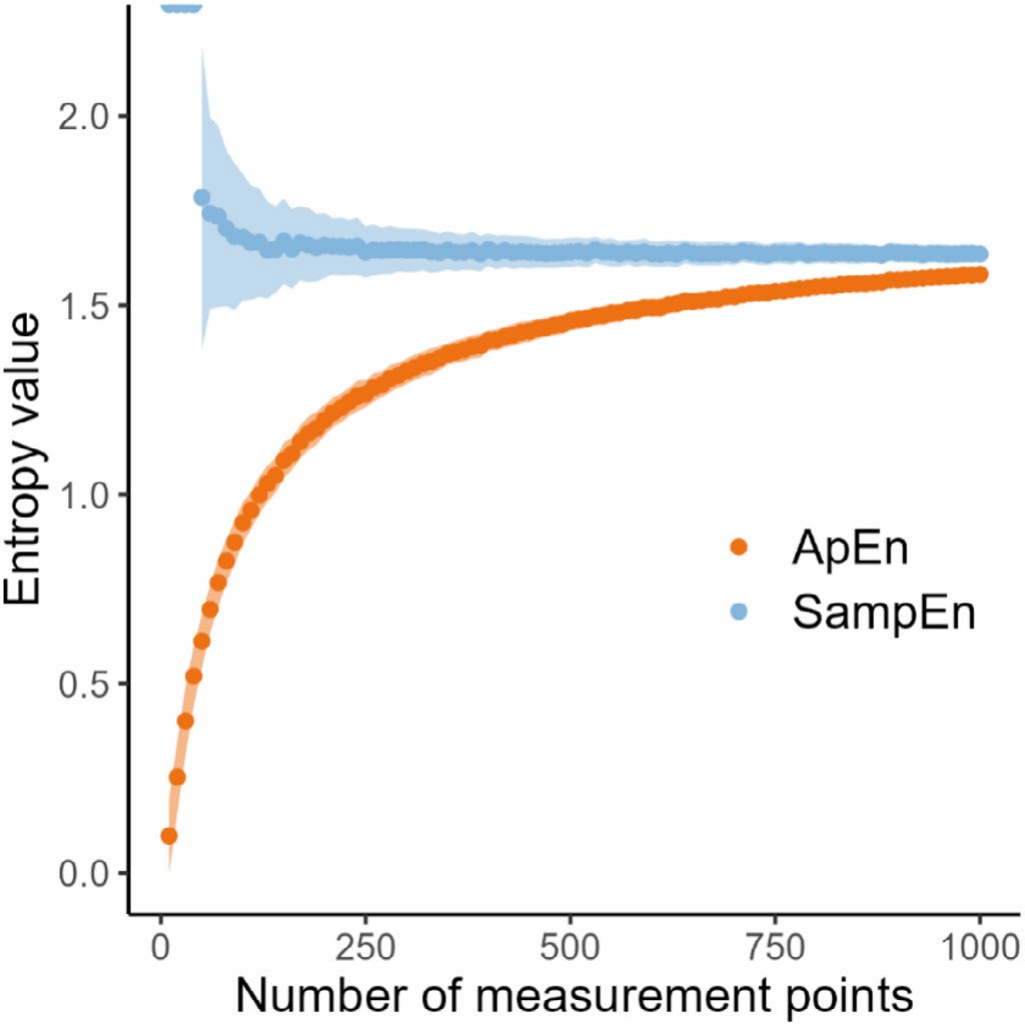
Values of ApEn and SampEn according to the number of respiratory cycles “*N*” in the simulations of VT. To generate this graph, we used similar methodology as Richman and Moorman (2000) but applied to our simulations. The effect of the number of respiratory cycles on the values of entropy is shown for one simulation (simulations of VT with a mean of 790 and a SD of 270) and using a variable number of simulated respiratory cycles. The value of SD had no influence on the ApEn and SampEn over “*N*” relationships, which was similar across all simulations (data not shown). Blue ribbons represent the SD of the SampEn measurement while orange ribbons represent the SD of the ApEn measurement.

Figure 6 shows the evolution of SampEn and ApEn when the number of respiratory cycles “*N*” is modified in the simulations. The value of ApEn is reduced with lower numbers of “*N*” while SampEn remains constant and lies close to the predicted value of entropy. On the other hand, the precision, represented by the SD of the measurement of SampEn, decreased, as represented by larger light blue ribbons, while the precision of ApEn remained constant (i.e., no wider light orange ribbons). Other simulations of VT, as well as simulations of BF using different dispersion values, yielded the same (ApEn – SampEn)/“*N*” relationship (data not shown).

### 3.6 Example in two real‐life examples focusing on the effect of the trends

**FIGURE 7:**
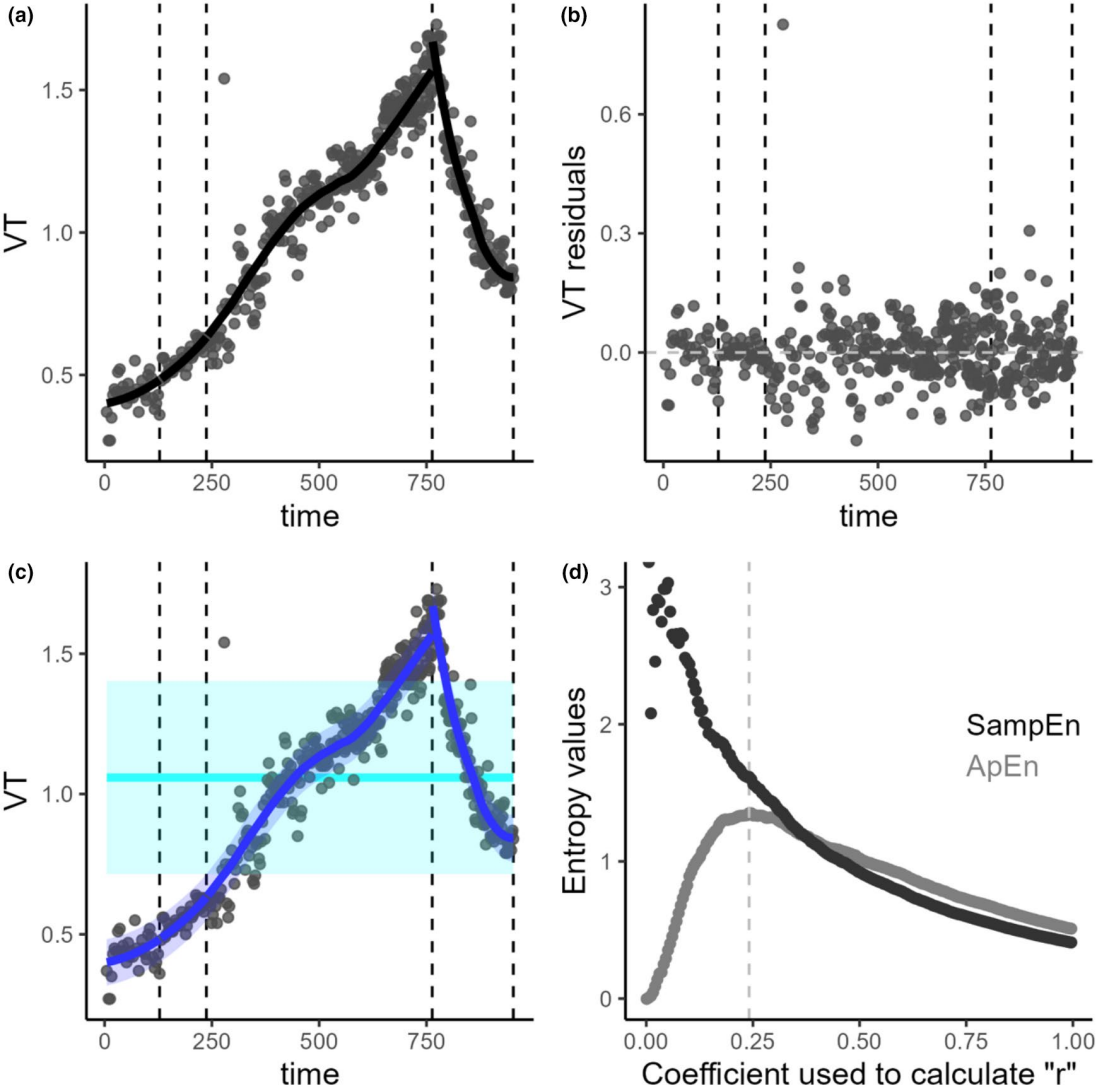
Real life example of the analysis of tidal volume from a patient with dysfunctional breathing with hyperventilation syndrome. Panel (a) shows the raw cycle‐by‐cycle data, with each grey point representing the VT value for a single respiratory cycle. The black line indicates the LOESS regression line. Panel (b) displays the residuals obtained from the LOESS regression. These residuals are generally centered around 0, with relatively stable variance. Panel (c) is similar to Panel (a) but illustrates the SD_trend_/SD_true_ ratio for this subject. The light blue line and light blue ribbon represent the mean and standard deviation (SD) respectively for the overall raw cycle‐by‐cycle data. The light blue ribbon corresponds to the SD_trend_ value. The dark blue line and dark blue line ribbon represent the LOESS regression line and the standard deviation around the trend (SD measured on the residuals), respectively. Thus, the dark blue ribbon represents the SD_true_ value. Panel (d) represents the ApEn and SampEn values according to the coefficient used to calculate the tolerance interval “*r*” for this individual. The grey line represents the coefficient that maximizes the value of ApEn (ApEn_max_). This specific individual had a total of 442 respiratory cycles (“*N*”). The values of ApEn_0.2_ and SampEn._0.2_ calculated on the raw cycle‐by‐cycle data were 0.71 and 0.65, respectively. The values of ApEn_0.2_ and SampEn._0.2_ calculated on the residuals of the LOESS were 1.31 and 1.74, respectively, (these values can also be visually estimated from panel (d) at a coefficient of 0.2). The coefficient that maximized the value of ApEn was 0.24 and the corresponding value of ApEn_max_ was 1.35. The SD_true_ was 0.072 L and the SD_trend_ was 0.344 L. The SD_trend_/SD_true_ value of this subject was at 4.18.

**FIGURE 8:**
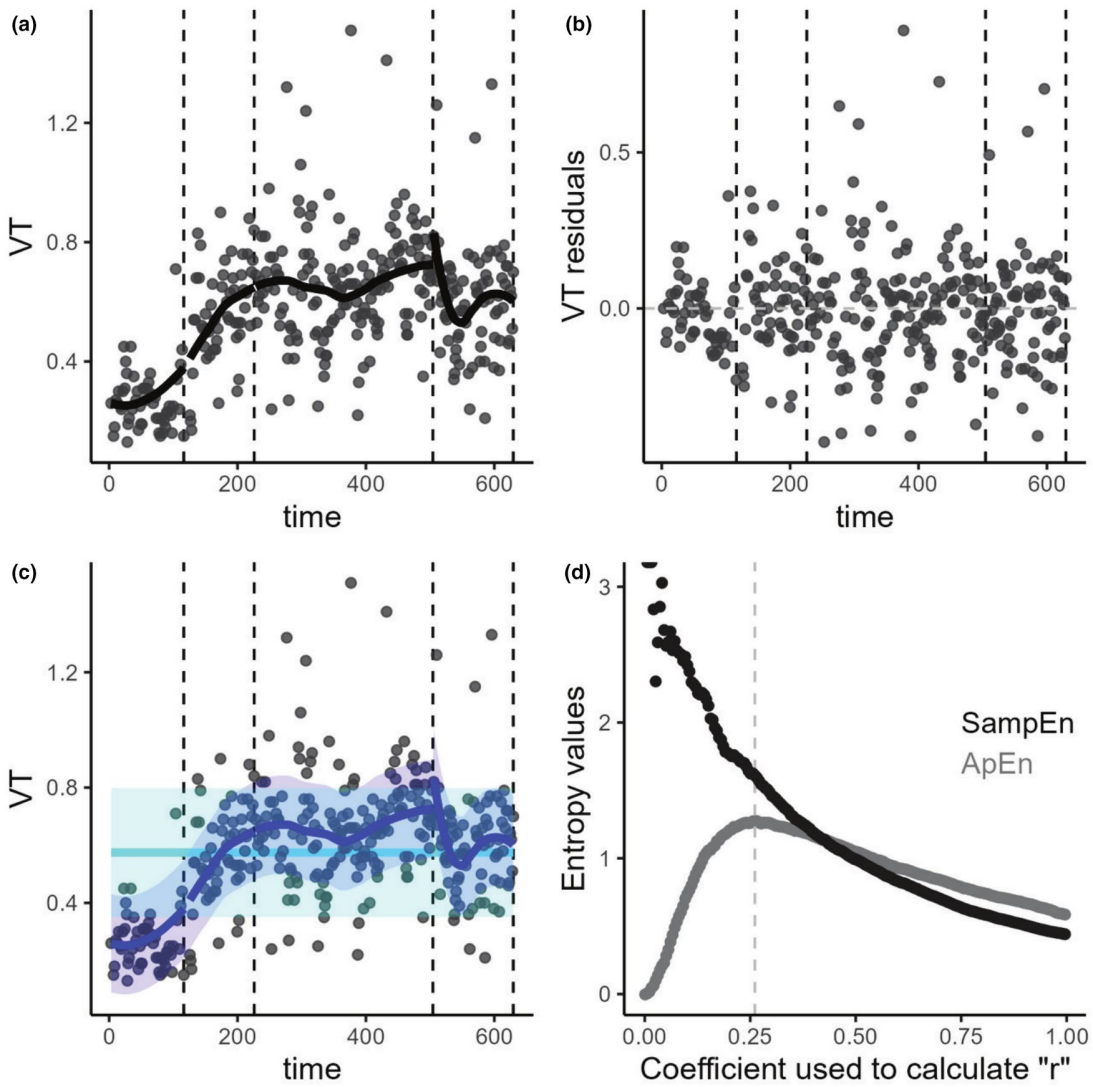
Real life example of the analysis of tidal volume from a patient with dysfunctional breathing and erratic breathing. Panel (a) shows the raw cycle‐by‐cycle data, with each grey point representing the VT value for a single respiratory cycle. The black line indicates the LOESS regression line. Panel (b) displays the residuals obtained from the LOESS regression. These residuals are generally centered around 0, with relatively stable variance. Panel (c) is similar to Panel (a) but illustrates the SD_trend_/SD_true_ ratio for this subject. The light blue line and light blue ribbon represent the mean and standard deviation (SD) respectively of the overall raw cycle‐by‐cycle data. The light blue ribbon corresponds to the SD_trend_ value. The dark blue line and dark blue line ribbon represent the LOESS regression line and the standard deviation around the trend (SD measured on the residuals), respectively. Thus, the dark blue ribbon represents the SD_true_ value. Panel (d) represents the ApEn and SampEn values according to the coefficient used to calculate the tolerance interval “*r*” for this individual. The grey line represents the coefficient that maximizes the value of ApEn (ApEn_max_). This specific individual had a total of 344 respiratory cycles (“*N*”). The values of ApEn_0.2_ and SampEn._0.2_ calculated on the raw cycle‐by‐cycle data were 1.17 and 1.65, respectively. The values of ApEn_0.2_ and SampEn._0.2_ calculated on the residuals of the LOESS were 1.20 and 1.76, respectively, (these values can also be visually estimated from panel (d) at a coefficient of 0.2). The coefficient that maximized the value of ApEn was 0.26 and the corresponding value of ApEn_max_ was 1.27. The SD_true_ was 0.155 L and the SD_trend_ was 0.223 L. The SD_trend_/SD_true_ value of this subject was at 1.31.

The effect of trend on entropy measurements is illustrated using two real‐life examples of patients undergoing CPET. The first example (Figure 7) represents a patient with a very small dispersion of VT around the trend and a normal rise of VT during exercise. The second example (Figure 8) shows a patient with high dispersion around the trend and a poor rise of VT. These two cases result in very different SD_trend_/SD_true_ ratios and correspondingly different magnitudes of bias in the entropy measurements when using trended versus detrended datasets.

In the first example, ApEn_0.2_ measured on the trended dataset is 0.71, while ApEn_0.2_ measured on the detrended dataset is 1.31. In the second example, ApEn_0.2_ on the trended dataset is 1.17, and ApEn_0.2_ calculated on the residuals is 1.20. The SD_trend_/SD_true_ ratio in the first case is 4.18, indicating a strong influence of the trend relative to the underlying signal dispersion. In contrast, the ratio in the second case is 1.31, which is close to 1, suggesting that the trend has minimal influence on the overall dispersion in the trended dataset.

This explains why, in the first case, we observe a large underestimation of the ApEn value when using the trended data compared to the detrended data, while in the second case, the difference is minimal. The first case typically represents the pattern seen in a healthy subject or a subject with pure hyperventilation syndrome, while the second case is classically representative of a subject with erratic breathing.

## 4 DISCUSSION

Dispersion and regularity statistics measure two distinct yet complementary concepts. Dispersion (e.g., SD, LOESS_0.75_) quantifies how far data deviate from their mean, while regularity statistics measure the predictability of data (Figure 1).

### 4.1 General comments about ApEn and SampEn measurements at rest

SampEn and ApEn values were similar across all simulations using a normal distribution of data and no trend (flat). The mean values of SampEn and ApEn for the data with no trend (flat) were 1.64 and 1.33, respectively, across all simulations (Tables 1 and 2).

We expected the entropy to remain constant across all magnitudes of dispersion because the entropy is a function of the probability distribution and not of the dispersion (Delgado‐Bonal & Marshak, 2019). Therefore, the entropy remained constant because:

1. We used a random process and a normal distribution of data that was similar across all simulations.
2. All parameters used to calculate the entropy for the simulations were constant (m = 2, *N* = 300, coefficient used to calculate “*r*” = 0.35 * SD). The tolerance interval “*r*” used to calculate ApEn or SampEn was set as a coefficient multiplying the SD of the data, which is standard practice when entropy is calculated (Pincus, 1991). In our case, the tolerance interval was set at 0.35 * SD because 0.35 was the coefficient that maximized the value of ApEn for the simulations.

Interestingly, the entropy calculation classically relies on the dispersion of the dataset using the SD in the calculation of the tolerance interval (Pincus, 1991). Therefore, it seems logical to always report the dispersion alongside the entropy calculation. This is also important because a given level of entropy is always interpreted in the context of a given dispersion. Indeed, similar values of ApEn and SampEn in the context of different dispersions (e.g., very low dispersion or very high dispersion) could represent drastically different physiopathological mechanisms. The different possible extreme combinations of entropy and dispersion are illustrated in Figure 1.

### 4.2 Effect of the trend of breathing parameters on entropy measurements and one potential solution to correct this effect

The effect of trends on the interpretation of Approximate Entropy (ApEn) was first highlighted more than 30 years ago by the creator of this method, who recommended correcting for any meaningful trends before interpreting entropy measurements (Pincus & Goldberger, 1994). Despite this recommendation, researchers have calculated entropy of breathing parameters during CPET without such correction (Bansal et al., 2018; Samaranayake et al., 2023). The calculation of ApEn and SampEn is based on comparing the distances (Y‐axis) between sequential points in a time series and evaluating the likelihood that these distances follow a predictable pattern (Pincus, 1991; Richman & Moorman, 2000). Therefore, from a mathematical standpoint, the theoretical entropy of a dataset should remain constant when a trend is applied. This would be true if “*r*” were a fixed value. However, in practice, the tolerance interval “*r*” is often defined as a coefficient multiplying the SD of the dataset. For example, in R statistical software, the tolerance interval is set by default at 0.2 * SD. The SD allows comparison of datasets with different dispersions (Pincus, 1991). However, for data with trends, the SD does not represent the dispersion of the data and is largely influenced by the trend (cf. Table 1). A trend will lead to a higher tolerance interval through “*r*” computation. A higher tolerance interval will allow more matches in the calculation of entropy, and more matches are interpreted as higher predictability (i.e., lower entropy) (Richman & Moorman, 2000). Therefore, calculating ApEn and SampEn on trended data using the uncorrected SD invariably leads to lower calculated entropy than predicted entropy (Figure 2 and Tables 1 and 2).

Overall, the effect of the trend on entropy was explained by the SD_trend_/SD_true_ ratio as shown in Figure 2, where the “SD_true_” represents the dispersion of the simulated data and the “SD_trend_” represents the value of SD calculated after a trend is applied. When a trend is applied, data are further scattered from the mean, and the SD always increases, influenced by both the dispersion of data and the trend. Higher SD_trend_/SD_true_ ratios, which represent a low dispersion of the data around the trended parameter combined with a high effect of the trend on the SD calculation, lead to lower entropy measurements. Using real‐life examples, this concept is illustrated in Figures 7 and 8.

Multiple methods exist to correct for trends. One way is to use the residuals obtained after computing the LOESS_0.75_ method. We previously demonstrated that LOESS_0.75_ was an appropriate method for estimating dispersion in simulated breathing parameters data during CPET, effectively illustrating the methods capability to detrend the data in most scenarios (Genecand et al., 2025). In this context, the entropy can be calculated on the dataset without trend using the SampEn calculated on the LOESS_0.75_ residuals and ApEn calculated on the LOESS_0.75_ residuals. As illustrated in Tables 1 and 2 and Figure 3, this method yielded entropy values very close to the predicted entropy. Importantly, the inaccuracies in SampEn calculated on the LOESS_0.75_ residuals and ApEn calculated on the LOESS_0.75_ residuals were explained by the inaccuracies of the LOESS_0.75_ method to estimate the dispersion and to detrend the data, as illustrated in Figure 4.

Other methods have been proposed to account for the trend of the dataset. For example, techniques such as differencing or detrending using Gaussian processes have been suggested and are implemented in one Python algorithm dedicated to entropy analysis (https://zblanks.github.io/eristropy/). Overall, we believe that multiple approaches can be valid for dealing with the stationarity issue of time series data. When using a detrending method for entropy calculations, authors should verify that residuals are approximately centered around zero and that the variance remains stable throughout the time series. Users should also be aware that all detrending methods involve a tradeoff: underfitting may lead to insufficient trend removal, while overfitting may result in the loss of meaningful signal (in the extreme case, overfitting would produce residuals reduced to a flat line at zero and a complete loss of signal). The superiority of one method over another to detrend breathing parameters during CPET has yet to be determined.

### 4.3 The ApEn‐SampEn/(coefficient of “*r*”) relationship and implications

Figure 5 shows the evolution of SampEn and ApEn when the coefficient used for the calculation of the tolerance interval “*r*” is modified. Entropy measurements should be lower when the tolerance interval is higher because more matches are accepted in the entropies calculation, and therefore data of a given dataset are considered more predictable (Pincus, 1991). SampEn exhibits logical behavior when the coefficient for calculating the tolerance interval is modified and represents an unbiased estimate of the true entropy measure (Richman & Moorman, 2000). Indeed, as shown in Figure 5, the lower the coefficient, the higher the entropy for SampEn. This is not true for ApEn. The relationship of ApEn over the coefficient used to calculate “*r*” has a bell‐shaped curve where the value of ApEn is maximal at a given value of the coefficient (Figure 5). Below this value, ApEn should increase when the coefficient decreases. Therefore, below the coefficient that maximizes entropy, ApEn yields a biased estimate of the true entropy (Richman & Moorman, 2000). We observed similar ApEn‐SampEn/(coefficient of “*r*”) relationships across all degrees of simulated SD showing that the magnitude of the dispersion had no impact on this relationship. This identical relationship, across all magnitudes of dispersion, is explained by the identical process used to generate the data (data randomly generated, following a normal distribution with the same length). Different types of datasets are expected to yield different ApEn‐SampEn/(coefficient of “*r*”) relationships, particularly the value of the coefficient that maximizes ApEn (Delgado‐Bonal & Marshak, 2019). When calculating ApEn, it has been suggested to use the coefficient of the tolerance interval that maximizes the value of ApEn, which allows us to estimate the greatest signal complexity (Delgado‐Bonal & Marshak, 2019). This has been evaluated both in simulations and real‐life data (Delgado‐Bonal & Marshak, 2019; Lu et al., 2008). For real‐life data, this usually represents an individual coefficient to calculate the tolerance interval for every patient. This is illustrated in Figures 7 and 8 where the coefficient that maximized ApEn in real‐life individuals is 0.24 and 0.26 respectively. Some have developed methods to automatically determine this coefficient (Chon et al., 2009). However, using a different coefficient for each patient makes comparisons between patients difficult. Another way to handle this problem would be to use SampEn with a fixed coefficient for the calculation of the tolerance interval (e.g., 0.2). Indeed, SampEn is an unbiased statistic of the true predictability when the coefficient used to calculate the tolerance interval is modified (Delgado‐Bonal & Marshak, 2019; Richman & Moorman, 2000).

Overall, the ApEn‐SampEn/(coefficient of “*r*”) relationship has major implications. First, we can see that for the same predictability of the data set, both values of entropy are significantly influenced by the coefficient used to calculate the tolerance interval. Therefore, authors should always report the parameter used for the calculation of the tolerance interval “*r*”. Additionally, it implies that studies using different coefficients for the tolerance interval calculation will not be comparable. If ApEn is measured, the coefficient that maximized its value should ideally be used (Delgado‐Bonal & Marshak, 2019). This will be potentially difficult since this coefficient will change for every patient analyzed and that a different coefficient for each patient makes comparisons between patients difficult. SampEn would be easier in this sense, since most fixed coefficients could be used to calculate “*r*” as long as the same coefficient is always used to compare different patients or the same patients at different times.

### 4.4 The ApEn‐SampEn/“*N*” relationship and implications

Figure 6 shows the evolution of SampEn and ApEn when “*N*” is modified. During CPET, the parameter “*N*” represents the number of respiratory cycles analyzed using the raw data. Patients undergoing CPET will have different numbers of respiratory cycles due to varying breathing frequency, exercise duration, and phases of the test. It is therefore of paramount importance to understand the influence of the parameter “*N*” on the measurement of SampEn and ApEn. In Figure 6, one can see that the value of ApEn decreases when “*N*” decreases despite similar predictability of the data. Because the predictability of the time series simulated is identical, one would expect the value of entropy to remain constant independently of the number of data points analyzed. This bias is explained by the inclusion of self‐matches in the calculation of ApEn, therefore artificially increasing predictability for small datasets (Richman & Moorman, 2000). In opposition to ApEn, SampEn follows the expected behavior, providing an unbiased estimate of entropy (Richman & Moorman, 2000). However, the lower bias of SampEn comes at the cost of diminishing precision with a lower number of “*N*” used. Overall, SampEn using classical parameters is not recommended for *N* < 200 points (i.e., number of respiratory cycles in our case) (Richman & Moorman, 2000; Yentes et al., 2013). With so few data points, SampEn has low precision. ApEn is known to be biased when *N* < 1000, often yielding lower‐than‐expected entropy values; however, its precision is preserved (Richman & Moorman, 2000). SampEn offers the advantage that individuals with a different number of respiratory cycles analyzed will be potentially comparable, while this will be more complicated with ApEn. Overall, the number of respiratory cycles analyzed should always be reported when using entropy measurements because this has a high impact on these measurements. This is especially important for CPET because the number of respiratory cycles obtained during this test lies in the critical zone of bias of entropy (data points often <1000).

Recently, authors have proposed an adaptation of SampEn using Bayesian optimization for small datasets (*N* < 200) (Blanks & Brown, 2024). Very low numbers of respiratory cycles (*N* < 200) are rarely observed during CPET when the recommended 8–12 min of exercise loading are met. Therefore, in most subjects, the use of classical entropy measures remains appropriate when analyzing complete CPET datasets, except in cases of premature test termination, which may warrant exclusion.

### 4.5 Examples using real‐life patients

The two real‐life cases shown in Figures 7 and 8 clearly illustrate how using entropy values on trended data can lead to the false interpretation that healthy subjects or subjects with DB with hyperventilation have lower entropy compared to subjects with erratic breathing. In the first example (Figure 7), the SD_trend_/SD_true_ ratio is very high, resulting in a significant underestimation of entropy when calculated on trended data. This occurs because, by default, when entropy is computed on trended data, the tolerance interval is based on the SD of the overall dataset (light blue ribbon, SD = 0.344 L). However, the actual signal complexity lies within a much smaller dispersion (dispersion around the trend, dark blue ribbon, SD = 0.072 L). This narrower underlying variability increases the number of matches during entropy computation, thereby lowering the entropy value. As a result, measuring entropy on trended data leads to a substantial underestimation of the true entropy in the case illustrated in Figure 7, an effect driven by the high SD_trend_/SD_true_ ratio.

The subject shown in Figure 8 is representative of patients with erratic breathing, which is characterized by a large dispersion of VT, sometimes associated with a poor rise in VT. The poor rise in VT and the larger underlying dispersion result in a lower SD_trend_/SD_true_ ratio (1.31), leading to only a small underestimation of entropy when using trended versus detrended data.

More interestingly, the two individuals reverse their relative entropy rankings depending on whether trended or detrended data is used. Based on trended data, one would conclude that the subject in Figure 7 has lower entropy than the subject in Figure 8. However, using detrended data, both subjects show very similar entropy, with the subject in Figure 7 actually exhibiting slightly higher entropy than the subject in Figure 8.

It is important to remember that when calculating entropy, the tolerance interval is derived from the SD of the dataset used for computation. For detrended data, in Figure 7, ApEn0.2 and SampEn0.2 are calculated using a tolerance interval of 0.072 * 0.2, while in Figure 8, the tolerance interval is 0.155 * 0.2. This means that the predictability signal for the subject in Figure 7 is assessed within an underlying dispersion that is approximately half that of the subject in Figure 8. This illustrates the key point that the entropy should always be analyzed in the context of the underlying dispersion, which can vary substantially in real‐life patients.

### 4.6 Summary of findings

Our study highlights several findings:

1. While the dispersion of the data around a parameter of interest is independent of regularity statistics, the opposite is untrue. Both SampEn and ApEn entropy calculations are usually computed using the underlying dispersion of data through the calculation of the tolerance interval, which is usually calculated as 0.2 * SD. Therefore, predictability is always interpreted in the context of a given dispersion. Describing the dispersion of data is essential because the same degree of unpredictability at different degrees of dispersion might represent drastically different physiological or pathological mechanisms.
2. Trends in ventilation data, reflecting a physiological response to exercise, lead to lower estimates of both SampEn and ApEn than expected. The effect of trends on entropy measurements depends on the ratio between the dispersion of data around the trend and the trend's impact on dispersion (SD_true_/SD_trend_).
3. Using the LOESS_0.75_ residuals is one way to calculate SampEn and ApEn after correcting for the trend induced by exercise. For entropy calculations, the users should always graphically visualize the data after detrend to evaluate whether these are centered on 0 with constant variance.
4. The number of respiratory cycles (“*N*”) and the coefficient used to calculate the tolerance interval (“*r*”) have a significant impact on entropy measurements and should be reported.
5. SampEn has advantages over ApEn because patients with different numbers of respiratory cycles “*N*” can be compared, and it allows the use of a constant coefficient for the calculation of the tolerance interval for all patients. Therefore, it might be the most promising regularity statistic in the field of exercise and DB. However, the precision of SampEn decreases when the number of respiratory cycles analyzed decreases. Therefore, SampEn is probably not suitable for datasets under 200 points (Yentes et al., 2013).

### 4.7 Future methodological considerations for entropy measurements

We think that other methodological points should be addressed in future studies in the field of entropy, CPET, and DB. These include:

1. Understanding the effect of outliers, such as sighs, on the values of entropy because this is a common phenomenon in patients with DB.
2. Investigating the effect of varying time intervals on the measurements of regularity statistics. Indeed, ApEn was developed for measurements equally spaced in time, and exercise is associated with an increase in breathing frequency and, therefore, an increase in the number of respiratory cycles for a given time.
3. Examining the effect of the evolution of the dispersion of the breathing parameter studied during exercise. Indeed, regularity statistics were developed for data with relatively stable variance over the time series, which is not always true during exercise.

### 4.8 Perspective of our findings with published studies using entropy in respiratory disease and DB patients

Breathing regulation is a complex process involving multiple physiological systems. Variability in respiratory patterns arises from several sources, including the respiratory central pattern generator, the chemical control system, phase resetting mechanisms, the limbic system's role in emotional regulation, cognitive influences, and voluntary control (Oku, 2022). Some studies have examined breathing parameters under resting conditions and across various disease states. These analyses have utilized both breath‐by‐breath data and high‐resolution time series. For instance, SampEn measured at rest in asthmatic patients was found to be lower in individuals with more severe airway obstruction (Veiga et al., 2011). In that study, high‐resolution respiratory flow data were recorded at a sampling rate of 16 Hz over 60 s, resulting in *N* = 960 data points analyzed per subject. Similar findings have been reported in patients with chronic obstructive pulmonary disease (Dames et al., 2014; Veiga et al., 2011). Conversely, individuals with panic disorder exhibit increased dispersion in breathing parameters, but entropy measures have seldom been measured in these patients (Grassi et al., 2013).

Entropy‐based metrics have been widely used to assess heart rate at rest and during exercise (Blanks et al., 2024; Pincus & Goldberger, 1994). In contrast, the analysis of breathing parameters and gas exchange using entropy during CPET has received relatively limited attention. Most analyses have traditionally relied on filtered, averaged panels and summary statistics considering unfiltered analysis as potential “noise”. However, with the growing interest in DB, unfiltered, breath‐by‐breath panels have been largely proposed and used to subjectively characterize DB patterns. In parallel, a few authors have begun applying entropy measurements during CPET to evaluate the complexity and variability of breathing parameters (Bansal et al., 2018; Blanks et al., 2024; Samaranayake et al., 2023).

Blanks et al. (2024) used a modification of entropy adapted to small datasets to analyze heart rate, gas exchange (breath‐by‐breath), and breathing parameters (breath‐by‐breath) during cardiopulmonary exercise testing (CPET) in 82 healthy children and adolescents aged 7–18 years. They found higher SampEn for heart rate in females compared to males and observed a decrease in heart rate SampEn before versus after the first ventilatory threshold (VT1) in both females and males. However, there were no significant changes in the SampEn of breathing frequency or tidal volume before versus after VT1, nor between males and females. The modification of SampEn used by Blanks et al may enable comparisons using smaller datasets and allow the analysis of different segments within a single CPET. However, for comparisons involving complete datasets in adults, individuals rarely have fewer than 200 breaths (*N* < 200) across the full duration of CPET. Therefore, classical SampEn measurement seems appropriate in this context (Yentes et al., 2013).

Two other studies investigated ApEn in subjects with DB compared to healthy controls during CPET, using cycle‐by‐cycle breath analysis (Bansal et al., 2018; Samaranayake et al., 2023). Notably, these studies did not apply trend correction to CPET data (Bansal et al., 2018; Samaranayake et al., 2023), a recommended step for entropy analysis (Blanks et al., 2024; Pincus & Goldberger, 1994; Richman & Moorman, 2000). Despite this, both studies reported meaningful discriminative results; it remains unclear whether trend correction would have modified these findings. Indeed, trends might themselves carry diagnostically relevant information. As shown in our two real‐life examples, patients with erratic breathing as compared to normal subjects or subjects with hyperventilation syndrome may exhibit distinct patterns in both the amplitude and progression of their breathing signals. This could lead to differences in the SD_trend_/SD_true_ ratio, potentially lower in erratic breathing patients, which might reduce the bias introduced by trends and result in higher entropy values.

### 4.9 General comments on various potential statistical techniques

A variety of techniques are available to quantify complexity in physiological signals, including dispersion and entropy. Simple measures such as the coefficient of variation (CV = SD/mean) and more advanced techniques like the root mean square of successive differences (RMSSD), autocorrelation analyses, detrended fluctuation analysis (DFA) for long‐term and short‐term correlations (LTC and STC), and the largest Lyapunov exponent (LLE) are among other available options. A comprehensive review of these techniques is provided by Oku Y. (Blanks & Brown, 2024; Oku, 2022). In this study, we focused on ApEn and SampEn, as they are specifically designed for short and noisy time series, which are typical of CPET data. In contrast, more general entropy measures, such as Kolmogorov–Sinai entropy, a generalization of Shannon entropy, are less suitable for such data (Delgado‐Bonal & Marshak, 2019). ApEn and SampEn have thus become the most widely used methods for assessing time series regularity in these conditions. Whether alternative approaches could provide better discriminative power or stronger correlations with patients' symptoms remains unclear and warrants further investigation.

### 4.10 Limitations

Our study has limitations. In addition to simulated data, we illustrated the effect of the trend on entropy measurements in only two real‐life patients. Using a larger number of real‐life patients to evaluate the effect of trends on entropy measurements should be the focus of future studies. Additionally, we applied LOESS_0.75_ to correct for the trend in exercise data; however, other methods may be more suitable for trend suppression.

## 5 CONCLUSIONS

SampEn and ApEn are influenced by the trend of breathing parameters during exercise tests. The residuals of the LOESS_0.75_ method can both estimate the dispersion of data around the trended parameters and provide a corrected estimate of ApEn and SampEn. Both the number of cycles analyzed and the coefficient used for the calculation of SampEn should be systematically reported. To compare patients with different numbers of respiratory cycles and for a more accessible measurement using a fixed parameter of the coefficient used to calculate the tolerance interval “*r*”, SampEn might be preferred over ApEn.

## AUTHOR CONTRIBUTIONS

L.G and C.J conceived the study. C.J designed and performed the statistical simulations. G.S and R.D verified the mathematical plausibility of all the simulations. L.G drafted the manuscript and handled the submission process. P‐O.B, I.F, M.A, and L.G analyzed the real‐life data of patients with post‐COVID‐19 DB that allowed for the statistical simulations. P‐O.B, F.L, C.J, A.Ber, I.G, C.C, R.D, G.S. I.F, M.A, A.Beu, P.L, D.M edited and re‐revised the first draft of the manuscript. All authors approved the final version of the manuscript.

## FUNDING INFORMATION

This study was supported by the Ligue pulmonaire genevoise, the Fondation Rankers Hartmann, the Ligue pulmonaire valaisanne, and foundation Lancardis.

## CONFLICT OF INTEREST STATEMENT

PL reports personal fees and support for attending meetings and/or travel from Chiesi, AstraZeneca, and GSK, outside of this work. None of the other authors has any conflicts of interest, financial or otherwise, to disclose in relation to this article.

## ETHICS STATEMENT

The real‐life parameters were based on a study involving human participants and were approved by (Commission cantonale d'éthique de la recherche sur l'être humain (CER VD), ID: 2021‐01698). Participants gave informed consent to participate in the study before taking part.

## PATIENT AND PUBLIC INVOLVEMENT

Patients and/or the public were not involved in the design, conduct, reporting, or dissemination plans of this research.

## Supporting information


Appendix S1.

## Data Availability

The data availability statement concerning the patients included in the case series is available elsewhere (Genecand et al., 2023). The statistical code allowing for the simulations is available in the Appendix S1 and free of use.
